# *SaRCC1*, a Regulator of Chromosome Condensation 1 (RCC1) Family Protein Gene from *Spartina alterniflora*, Negatively Regulates Salinity Stress Tolerance in Transgenic *Arabidopsis*

**DOI:** 10.3390/ijms23158172

**Published:** 2022-07-25

**Authors:** Wanchang Li, Jian Wen, Yueyi Song, Huiyan Yuan, Bin Sun, Ren Wang, Sheng Xu

**Affiliations:** 1 College of Life Sciences, Henan Normal University, Xinxiang 453007, China; li_wan_chang@163.com (W.L.); vincent_1516@163.com (J.W.); 2 Institute of Botany, Jiangsu Province and Chinese Academy of Sciences, Nanjing 210014, China; yuanhy422203@163.com (H.Y.); sunbin9825@163.com (B.S.); rwang@cnbg.net (R.W.); 3 Institute of Virology and Biotechnology, Zhejiang Academy of Agricultural Sciences, Hangzhou 310021, China; 4 College of Landscape Architecture, Nanjing Forestry University, Nanjing 210037, China; songyueyi2021@163.com

**Keywords:** regulator of chromosome condensation 1, salinity stress, *Spartina alterniflora*, *UVR8*

## Abstract

A regulator of chromosome condensation 1 (RCC1) family protein has been functionally characterized to be involved in various cellular processes. In this study, one *RCC1* gene named *SaRCC1* was cloned from the full-length cDNA library of *Spartina* *alterniflora*. The open reading frame (ORF) of *SaRCC1* was 1440 bp, and it encoded 479 amino acids with a calculated molecular mass of 51.65 kDa. Multiple amino acid sequence alignments showed that SaRCC1 had high identity with other plant RCC1s, and the phylogenetic analysis indicated that SaRCC1 had a closer affinity to *Zea mays* RCC1 family protein (ZmRCC1). *SaRCC1* gene was induced under salt stress conditions, and its encoded protein was located in peroxisome. In order to further investigate the function of *SaRCC1*, transgenic *Arabidopsis* plants ectopically both sense-overexpressing and antisense-overexpressing *SaRCC1* were generated. *SaRCC1*-overexpressing lines exhibited an increased salt and ABA hypersensitivity and reduced resistance to salinity stress. On the other hand, the transcripts of some stress-responsive genes in the *SaRCC1* transgenic plants were affected in response to salinity stress. Our results provide evidence for the involvement of *SaRCC1*, negatively regulating salt stress responses by affecting stress-related gene expression in *Arabidopsis*.

## 1. Introduction

Salinity is a major environmental stress in nature limiting plant growth and productivity. It affects many aspects of plants by imposing several major constraints including osmotic stress, ionic imbalance, and secondary stress [1,2]. Plants must have evolved a plethora of mechanisms, such as the ability to sense salt stress, to transduce signals to cell interiors, to adjust cellular traits, and to avoid the damage caused by the soil high salt concentrations in [2,3]. However, the ability of plants to tolerate salt stress varies between and within species, and because of the complexity, the genetic control of plant salt tolerance has not yet been fully understood [1,2].

The regulator of the chromosome condensation 1 (RCC1) family protein is regarded as a critical cell cycle regulator. It is the guanine nucleotide exchange factor for Ran, a Ras-like nuclear GTPase, and is usually made of a seven-bladed β propeller structure, constituting the RCC1 domain [4]. RCC1 family proteins could be directly involved in the assembly of mitotic spindles, formation of the nuclear envelope, transport of nuclear material, and regulation of cell cycle G1/S transition. Besides, numerous studies have revealed that they also play important roles in tumor biology [5,6]. In plants, RCC1 family proteins could act as regulating factors for mediating diverse biological processes such as the ultraviolet-B (UV-B) radiation response, abscisic acid (ABA) signaling, cold tolerance, and phenotypic plasticity [7,8,9,10]. The most studied and sole plant representative of the RCC1 family protein is UV-B RESISTANCE LOCUS 8 (UVR8), the UV-B photoreceptor, which is responsible for affecting a series of self-protection genes’ expression under UV-B, thereby evoking many different physiological responses [11,12,13]. UVR8 also participates in the response to multiple stresses, and its transcript expression could be up-regulated by chilling, salinity, and osmotic stress [13,14]. For example, the expression of *Arabidopsis UVR8* was slightly increased under different abiotic stresses, except for the important increment with osmotic and salt stress treatments [14,15]. RCC1/UVR8/GEF-like 3 (RUG3) is another RCC1 family protein, which interacts with ataixa-telangiectasia mutated (ATM) in mitochondria to synergistically regulate splicing of the *nad2* mRNA and complex I biogenesis, which is essential for ROS homeostasis and plant development [16,17]. RUG3 also acts as a negative regulator of plant responses to ABA [18]. In addition, RCC1 family protein gene *TCF1* (*Tolerant to Chilling and Freezing 1*) regulates plant tolerance to a low temperature in a C-repeat binding transcription factor (CBF)-independent pathway [8,19]. Recently, the RCC1 family protein SAB1 (Sensitive to ABA 1) was identified as a crucial component of ABA signaling by negatively regulating ABI5 (ABA-insensitive 5) through multiple mechanisms to modulate early plant development [9]. Moreover, the RCC1 family protein PROTON1 (PLASTICITY OF ROSETTE TO NITROGEN 1) was identified to be involved in controlling the plasticity of the rosette diameter in response to nitrogen [10]. However, no functional analysis of the plant RCC1 family protein involved in salinity stress has been investigated, except for UVR8 [15].

As a perennial deciduous grass and halophyte, *Spartina alterniflora* (Loisel.) has been regarded as a useful model for mining candidate genes involved in plant salt tolerance [20]. Because halophytes have specialized molecular mechanisms or special cellular structures to tolerate high salt concentrations, underlying knowledge of the molecular mechanisms for adaption or tolerance to salt stress in halophytes could provide potential application in crops to improve their capacity to accumulate high concentrations of ions. In a previous study, cDNA library profiles of *S. alterniflora* under salt treatment were constructed to identify the potential key genes involved in the response to salt stress [21]. In this study, we identified an RCC1 family protein gene *SaRCC1*, and its detailed gene expression patterns, subcellular localization, as well as function analysis were investigated. Our results showed that *SaRCC1* might negatively affect plant salt stress tolerance.

## 2. Results

### 2.1. Characterization and Sequence Analysis of SaRCC1

**Figure 1:**
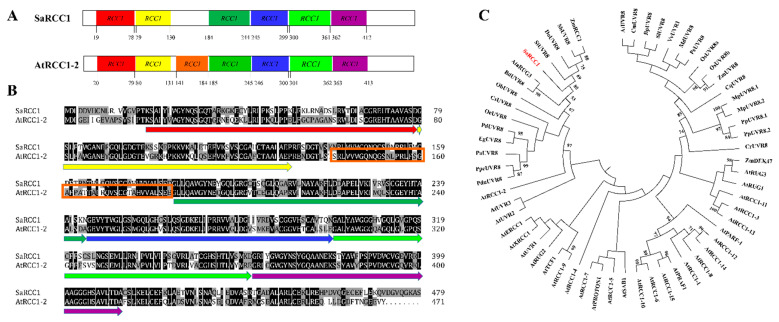
The SaRCC1 protein structure, sequence alignments, and phylogenetic relationships of *SaRCC1* and other plant RCC1 family proteins. (**A**) Computational analyses of SaRCC1 protein. Expasy online software (http://www.prosite.expasy.org/scanprosite, accessed on 6 May 2022) predicted that *Arabidopsis* RCC1 family protein AtRCC1-2 (At3g02300) contains seven RCC1 repeats, whereas SaRCC1 protein contains six predicted RCC1 repeats. (**B**) Amino acid alignment analysis of identity between AtRCC1-2 and SaRCC1 protein. Arrows and box in different colors mark RCC1 repeat domains. (**C**) The phylogenetic relationships of SaRCC1 and its closely related RCC1 family proteins in other plants. The phylogenetic tree was constructed with MEGA version 5.0 software, and numbers on branches indicate the percentage of bootstrap analysis supporting the grouping of each branch. SaRCC1 is highlighted in red.

Of the sequenced clones in the full-length cDNA library created from the salt-treated *S.* *alterniflora* seedlings [21]. a full-length cDNA sequence with high homology with the RCC1 family protein gene was obtained and selected for further study. This gene comprises a 121 bp 5′-untranslated region (UTR), 1440 bp ORF encoding a 479-amino acid residue protein, and a 298 bp 3′-UTR (Figure S1). It encodes an RCC1 family protein containing six predicted tandem RCC1 repeats; sequence alignments indicate that *SaRCC1* shares 66.7% identity in ORF sequence and 70.5% identity in amino acid sequence with *Arabidopsis* RCC1 family protein (At3g02300, here named *AtRCC1-2*), suggesting that these two are likely orthologs (Figure 1A,B and Figure S2). The molecular mass of SaRCC1 is about 51.65 kDa, and its isoelectric point value is 6.49.

Sequence alignments by BLASTP in the NCBI database indicated that SaRCC1 shares a high level of sequence identity with other RCC1 proteins (including predicted UVR8) from *Sorghum bicolor* (SbUVR8, XP_021307437.1; 92.36%), *Zea mays* (ZmRCC1, NP_001159191.1; 92.57%), *Setaria italica* (SiUVR8, XP_004982469.1; 91.23%), and *Dichanthelium oligosanthes* (DoUVR8, OEL17715.1; 91.30%). Besides, a phylogenetic tree was constructed to analyze the relationships between SaRCC1 and other plant RCC1 domain-containing proteins (Table S2), and the result showed that SaRCC1 proteins were closely related to SbUVR8, SiUVR8, DoUVR8, and ZmRCC1 (Figure 1C).

### 2.2. Expression Analysis of SaRCC1 in Response to NaCl Stress

**Figure 2:**
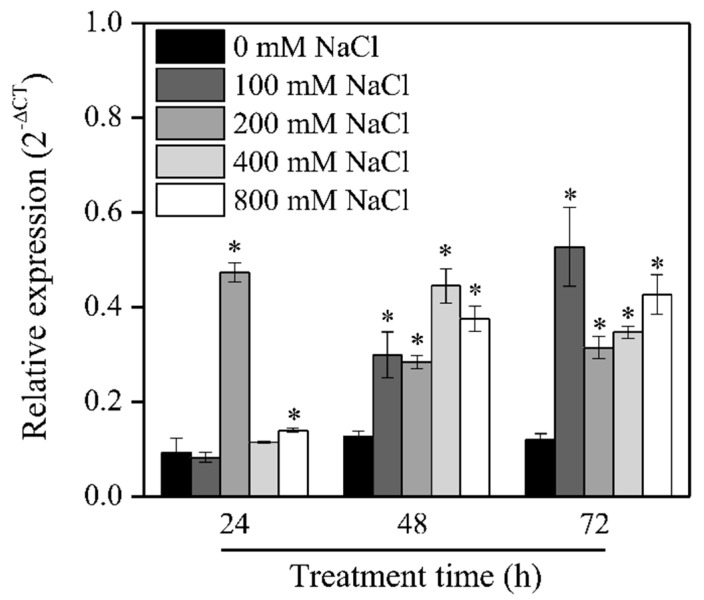
Expression pattern of *SaRCC1* gene under salinity stress. *S. alterniflora* seedlings were subjected to different concentrations of NaCl, and the leaves were then collected for *SaRCC1* gene expression analysis at the indicated time points. *SaTubulin* was used as the reference gene. The asterisks over the bars indicate statistical significance (*p* < 0.05) at each treatment time.

qRT-PCR was performed to examine the expression level of *SaRCC1* in *S. alterniflora* seedlings under salinity stress conditions. *SaRCC1* transcripts were increased in *S. alterniflora* seedlings treated with different concentrations of NaCl (Figure 2). For example, after 48 h of treatment with 400 mM NaCl, the mRNA level of *SaRCC1* reached the maximum, and was about 3.5-fold higher than that in the control (0 mM NaCl) (Figure 2). The result suggests that the expression of *SaRCC1* is responsive to salt treatment.

### 2.3. Subcellular Localization Analysis

**Figure 3:**
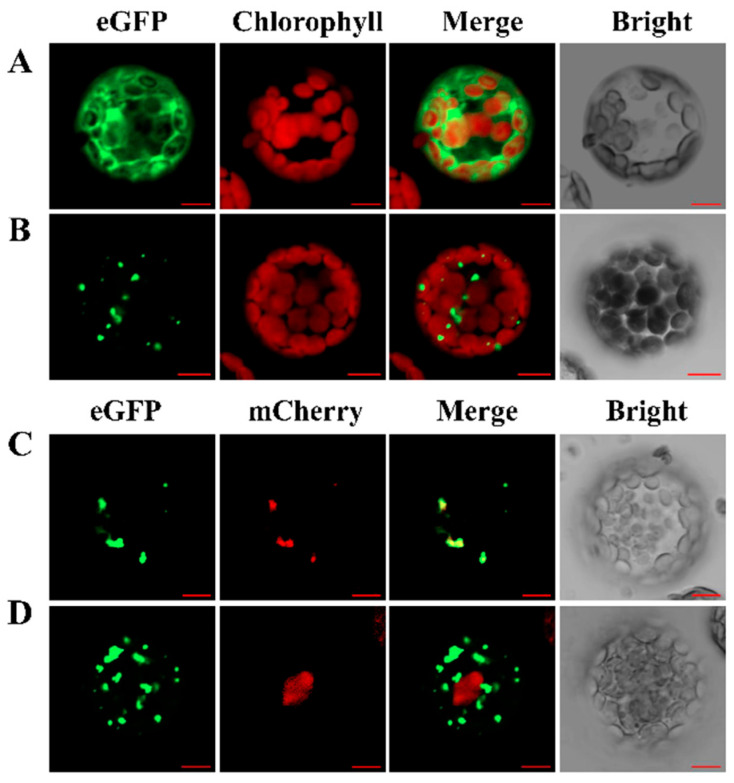
Localization of SaRCC1–eGFP fusion protein. eGFP alone (**A**) or SaRCC1–eGFP fusion protein (**B**) expressed under the control of the CaMV 35S promoter in *Arabidopsis* protoplasts. (**C**) *Arabidopsis* protoplasts co-expressing SaRCC1–eGFP and AtHMGB1–mcherry protein. (**D**) *Arabidopsis* protoplasts co-expressing SaRCC1–eGFP and AtPEX7–mcherry protein. The pictures were photographed under the green channel (eGFP fluorescence), red channel (mCherry fluorescence), combination of the green and red channels, and bright channel. Chlorophyll represents chlorophyll autofluorescence. Scale bar = 10 μm.

The eGFP reporter gene construct was fused to *SaRCC1* under the control of the cauliflower mosaic virus (CaMV) 35S promoter, and the *Arabidopsis* protoplasts transiently expressed the fusion protein vector 35S: SaRCC1–eGFP via PEG-mediated transformation to experimentally determine the subcellular localization of SaRCC1 protein. As shown in Figure 3A, the fluorescence signal of eGFP itself was visualized throughout the cell (Figure 3A), whereas the fluorescence signal from SaRCC1–eGFP fusion protein was detected in a punctate pattern (Figure 3B), and was generally identical to those of the red fluorescence of AtPEX7–mcherry fusion protein (Figure 3C). As AtPEX7 has been clearly reported to be a peroxisomal protein receptor [22]. our result showed that SaRCC1 is very likely to be localized in peroxisome.

### 2.4. The Characteristics of SaRCC1 Sense-Overexpressing and Antisense-Overexpressing Transgenic Arabidopsis

To determine the function of *SaRCC1*, *Arabidopsis* plants were transformed to express sense or antisense *SaRCC1* under the control of CaMV 35S promoter (Figure S4A). For simplicity, the transgenic plants from the vector containing *SaRCC1* antisense RNA were called anti-*SaRCC1*. Five and ten independent transformed lines for sense *SaRCC1* and Anti-*SaRCC1*, respectively, were thus produced. The genomic DNAs were isolated from *Arabidopsis* wild-type (WT) plants and transgenic lines and validated by amplifying the hygromycin resistance gene (*HPT II*). The expected 697 bp PCR fragment was amplified from the DNA of transgenic lines, but not from those of WT plants (Figure S4B). T_3_ homozygous transgenic lines were obtained for *SaRCC1* expression analysis. From the result of semi-quantitative reverse transcription PCR (RT-PCR), the expression of *SaRCC1* was confirmed from the transformant, but not from the non-transformed control (Figure S4C). In addition, the expressions of *AtUVR8* (At5g63860) and *AtRCC1-2* were also analyzed in WT and *SaRCC1* transgenic *Arabidopsis* plants, and no dramatic changes were observed (Figure S4C). Finally, four homozygous lines (#−2, #-(#−3, #-(#−8, and #-(#−10) with *SaRCC1* antisense-overexpressed in transgenic plants and three homozygous lines (#3, #4, and #5) with SaRCC1 sense-overexpressed in transgenic plants were selected for further analysis.

**Figure 4:**
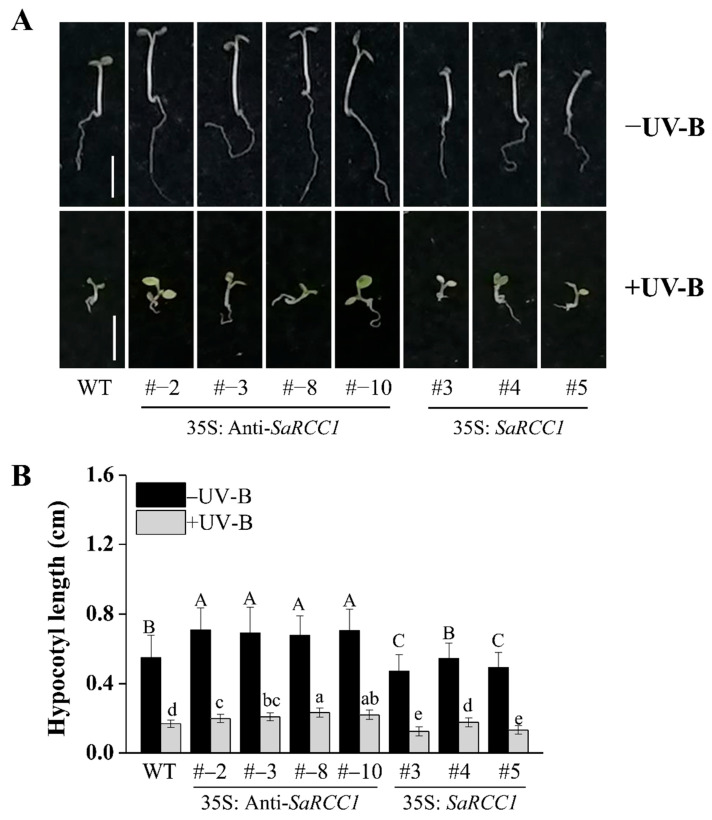
Phenotypes (**A**) and hypocotyl length (**B**) of WT and *SaRCC1* transgenic seedlings grown under white- and UV-B-light conditions for 5 days. Scale = 0.5 cm. Different letters indicate significant differences (*p* < 0.05) according Duncan’s multiple test.

UVR8 is a positive photoreceptor that inhibits hypocotyl growth [23]. As phylogenetic analysis also showed that SaRCC1 was clustered with the predicted UVR8 from some plant species, the potential function of *SaRCC1* in regulating UV-B-induced photomorphogenesis characterized by the inhibition of hypocotyl growth was examined. As shown in Figure 4, under white light conditions, significant differences among hypocotyl length of WT, *SaRCC1*-ovexpressing, and *SaRCC1*-anitsense transgenic lines were observed. When grown under white light supplemented with narrowband UV-B light, the hypocotyl growth of all the seedlings was dramatically inhibited. Among them, *SaRCC1*-anitsense transgenic lines exhibited reduced inhibition of hypocotyl elongation compared with WT, while the hypocotyl length of the *SaRCC1*-ovexpressing lines was the shortest.

### 2.5. Overexpression of SaRCC1 in Arabidopsis Plants Sensitive to Salinity Stress

**Figure 5:**
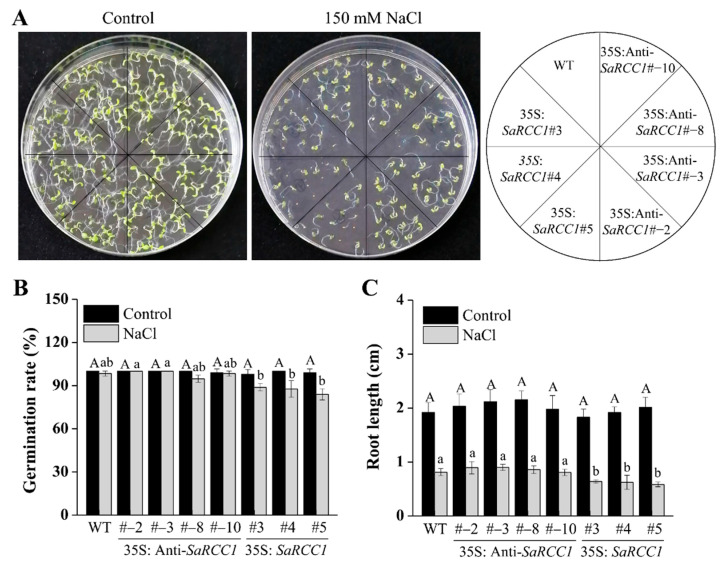
Analysis of salinity tolerance in *Arabidopsis* wild-type (WT) and *SaRCC1*-overexpressing and antisense-overexpressing lines. The phenotype (**A**), germination rate (**B**), and root length (**C**) of wild-type (WT) and *SaRCC1* overexpressing and antisense-overexpressing *Arabidopsis* on 1/2 MS medium supplemented with or without 150 mM NaCl for 7 d. Different letters indicate significant differences (*p* < 0.05) according Duncan’s multiple test.

To study the potential role of *SaRCC1* in salt stress response in transgenic *Arabidopsis* plants, seed germination and seedling growth (root length) were evaluated in WT and transgenic *SaRCC1* lines with NaCl stress treatments. Under normal growth conditions (on 1/2 MS medium), the seed germination of *SaRCC1* transgenic lines had no significant difference from WT (Figure 5A). However, under the treatment with 150 mM of NaCl, far greater germination inhibition occurred than what was observed in *SaRCC1*-overexpressing lines when compared with the WT control (Figure 5A,B). Under normal growth conditions, the root lengths did not significantly differ among WT and *SaRCC1* transgenic plants (Figure 5C). However, the primary root lengths of the *SaRCC1*-overexpressing lines were significantly shorter than those of the WT plants under salinity stress (Figure 5C). On the other hand, no obvious differences were observed between *SaRCC1*-antisense transgenic lines and WT plants in terms of both germination rate and root growth. The above results indicated that overexpressing *SaRCC1* are sensitive to salinity in transgenic *Arabidopsis*.

### 2.6. Transgenic Plants Overexpressing SaRCC1 Are Hypersensitive to ABA

**Figure 6:**
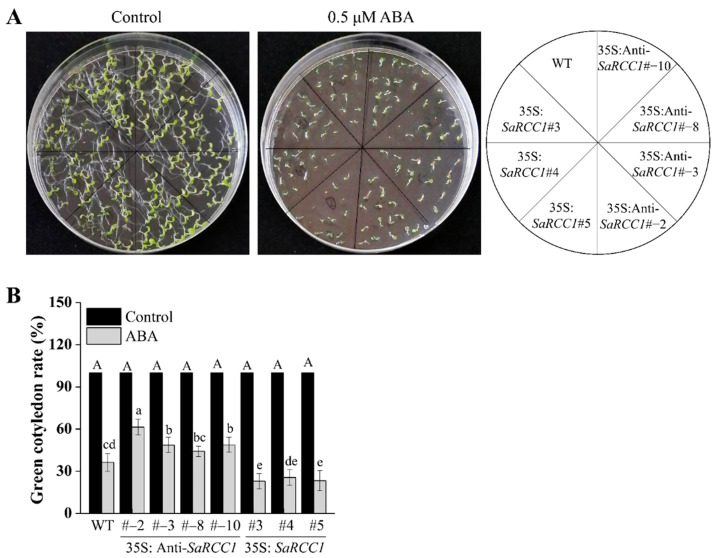
Phenotypes of wild-type (WT) and *SaRCC1* transgenic *Arabidopsis* lines under abscisic acid (ABA) treatment. (**A**) Seedling growth of WT and *SaRCC1* transgenic *Arabidopsis* lines on 1/2 MS medium supplemented with or without 0.5 μM of ABA after incubation for 7 days. (**B**) Green cotyledon rate of WT and *SaRCC1* transgenic *Arabidopsis* lines. Different letters indicate significant differences (*p* < 0.05) according Duncan’s multiple test.

ABA signaling is frequently affected by salinity stresses in plants [2]. The responses of the *SaRCC1* transgenic lines under exogenous ABA treatment during seed germination and post-germination growth were subsequently examined. When grown on 1/2 MS medium, seedling emergence of *SaRCC1* transgenic plants had no significant difference from WT (Figure 6A). However, the percentage of *SaRCC1*-overexpressing transgenic plant seedlings with green cotyledons was significantly lower than that of WT when treated with 0.5 μM ABA, whereas the percentage of *SaRCC1* antisense-overexpressing transgenic plant seedlings with green cotyledons was significantly higher than that of WT (Figure 6A,B). Furthermore, *SaRCC1* transgenic plants were subjected to 200 mM mannitol for osmotic stress. Under normal growth and osmotic stress conditions, the germination rate and seedling growth were not significantly different among WT, *SaRCC1*-overexpressing, and *SaRCC1*-antisense-overexpressing lines (Figure S5). However, the *SaRCC1*-antisense transgenic seedlings exhibited less growth inhibition than WT under 200 mM mannitol treatment (Figure S5C,D).

### 2.7. The Effects of SaRCC1 on the Expression of Stress-Responsive Genes

**Figure 7:**
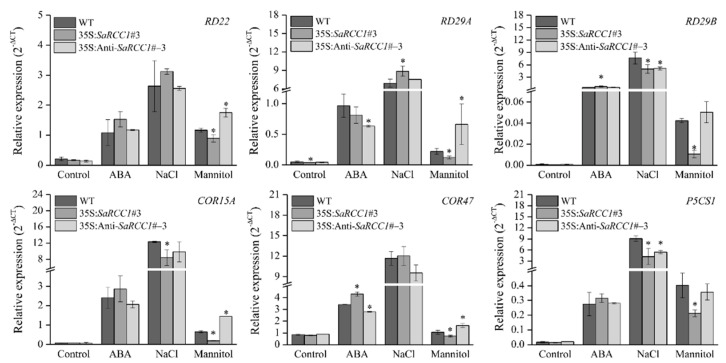
Expression levels of stress-responsive genes in *Arabidopsis* wild-type (WT) and transgenic plants under salinity stress, ABA treatment, and osmotic stress conditions. Seven-day-old seedlings were subjected to 100 mM NaCl, 5 μM ABA, or 300 mM mannitol, respectively, for 24 h, before determining the expression levels of *RD22*, *RD29A*, *RD29B*, *COR15A*, *COR47*, and *P5CS1* by qRT-PCR. *Actin2* was used as an internal reference gene. The asterisks over the bars indicate the significant differences between the wild-type and *SaRCC1* transgenic *Arabidopsis* lines.

To investigate the potential molecular mechanism involved in the regulation of *SaRCC1* under salinity stress conditions, a quantitative expression analysis of six stress-associated genes, including *RD22*, *RD29A*, *RD29B*, *COR15A*, *COR47*, and *P5CS1*, in transgenic *Arabidopsis* and WT plants was conducted (Table S1). As expected, *SaRCC1* genes were highly expressed in transgenic *Arabidopsis* plants, whereas *AtUV*R8 and *AtRCC1-2* basically showed no obvious difference among WT, *SaRCC1*-overexprssing, and *SaRCC1*-antisense-overexpressing transgenic plants under both normal and stress conditions (Figure S6). In addition, under normal growth conditions, the expression levels of *RD22*, *RD29B*, *COR15A*, *COR47*, and *P5CS1* did not exhibit obvious differences among WT, *SaRCC1*-overexpressing, and *SaRCC1*-antisense-overexpressing lines. However, when treated with salinity, ABA, or mannitol, the expression levels of all of the tested genes were rapidly induced; among them, the expression levels of *RD29B*, *COR15A*, and *P5CS1* genes were lower in the *SaRCC1*-overexpressing seedlings than WT under salinity stress (Figure 7). In addition, with mannitol treatments, the expression of *RD22*, *RD29A*, *RD29B*, *COR15A*, and *COR47* was lower in the *SaRCC1*-overexpressing seedlings than WT, while the opposite trends were observed in *SaRCC1*-antisense transgenic plants when compared with WT plants (Figure 7).

## 3. Discussion

In many eukaryotes, the RCC1 motif has been defined as a conserved domain consisting of 51–68 amino acid residues to fold into a bladed beta-propeller [24]. RCC1 family proteins were first characterized to function in the regulating cell cycle, but since then, different members of this family have been identified to have diverse functions [4]. In animals, RCC1 is critical for activating Ran and is involved in various biological processes coupled with Ran [4,5,6]. In plants, the RCC1 family proteins could be grouped into two major categories: one consisting of 6–7 RCC1 repeats similar to human RCC1, and the other composed of multiple domains, including the RCC1 repeats domain [16]. Despite that emerging evidence has showed that the plant RCC1 family protein mediates different biological processes [8,9,10,16,17,18,25], its detailed biological role in plants remains largely unclear. For example, *Arabidopsis* contains 24 putative RCC1 family proteins, but only five of them (UVR8, TCF1, RUG3, SAB1, and PROTON1) have been functionally characterized [8,9,10,16,17,18,24]. In maize, 31 putative RCC1-like proteins are identified, while only the functional role of ZmUVR8 in UV-B signaling [26] and DEK47 in kernel development [23] has been revealed. In upland cotton, by searching for the associated reference genomes, 56 RCC1 family protein genes were characterized and most of them are expressed differently under various hormone treatments and abiotic stress [27]. Among them, only two RCC1 family protein genes (Gh_A05G3028 and Gh_D10G2310), which are homologous to UVR8, were functionally analyzed [27].

In this study, the molecular cloning, expression, and functional identification of *S.* *alterniflora* RCC1 family protein gene (*SaRCC1*) were characterized. Sequence analysis showed that the deduced SaRCC1 protein contained six typical RCC1-like domains and was highly conserved with other plant RCC1 family proteins, confirming that SaRCC1 shares the typical characteristics of RCC1 proteins (Figure 1). Although phylogenetic analysis also showed that SaRCC1 was clustered with some plant UVR8 annotated in NCBI, it still shares a low homolog with the functionally identified UVR8, such as AtUVR8 (24.5%, Figure 1C and Figure S3, Table S2), suggesting that SaRCC1 might not function as a canonical UV-B photoreceptor.

The different subcellular localization of RCC1 family proteins has been observed: UVR8 localizes to both the cytosol and nucleus; however, with UV-B irradiation, UVR8 monomers tend to accumulate in the nucleus, and constitutive photomorphogenic 1 (COP1) plays a dual role in UVR8 nuclear accumulation and downstream signaling [13]. Similar to UVR8, the dual subcellular localization of RCC1 family protein SAB1 in both cytosol and the nucleus is also observed [9]. However, unlike UVR8, TCF1 is exclusively localized in the nucleus [8,19]. and RUG3 localizes in mitochondria [16,17,18]. We experimentally confirmed that SaRCC1 is mainly localized in the peroxisome, showing a totally different subcellular localization pattern to the characterized RCC1 proteins (Figure 2). Neither did we observe any redistribution of SaRCC1 protein to the nucleus under UV-B treatment (data not shown).

RCC1 family protein genes were also identified as stress-responsive in different plant species, and most of them are expressed differently under abiotic stress and various hormone treatments [13,14]. We also observed that *SaRCC1* was significantly induced under NaCl stress treatments (Figure 3). In this study, to understand the possible biological role of *SaRCC1*, transgenic *Arabidopsis* lines overexpressing sense or antisense *SaRCC1* RNA were constructed. As one of the antisense technologies, antisense expression of a target gene can serve as a tool to study the effect of a particular plant gene inactivation and the functional role of genes in plant diverse processes [28,29]. For example, antisense expression of pectate lyase gene or β-galactosidase gene alters the softening of strawberry fruit [30,31]. Antisense expression of an *Arabidopsis* ω-3 fatty acid desaturase gene reduces salt/drought tolerance in transgenic tobacco plants [32]. whereas transgenic tobacco expressing antisense apple TFL1-like gene (*MdTFL1*) exhibited distinct morphological changes in lateral shoot outgrowth; internode length; and the development of leaves, flowers, and fruits [33]. We also noticed that *SaRCC1*-ovexpressing transgenic *Arabidopsis* exhibited a shorter hypocotyl length, while 35S: anti-*SaRCC1* plants exhibited a longer hypocotyl length compared with WT seedlings under both weak white light and UV-B irradiation conditions, suggesting that *SaRCC1* might be involved in regulating photomorphogenesis (Figure 4). However, considering the low sequence identity between SaRCC1 and AtUVR8, whether SaRCC1 participates in UV-B-induced photomorphogenesis should be further investigated.

With 150 mM NaCl treatments, when compared with WT *Arabidopsis*, the *SaRCC1*-overexpressing lines were reduced in their capacity of salt tolerance in seed germination and seedling growth (Figure 5), confirming its potential negative role in response to salt stress. Phytohormone ABA plays an important role in a wide range of biological processes, such as plant development and response to abiotic stresses, and it is mainly involved in the regulation of salt stress responses, including stomatal closure, ion homeostasis, metabolic change, and salt-stress-responsive gene expression [2]. We also show that *SaRCC1* plays a role in ABA-mediated regulation of seed germination. The overexpression of *SaRCC1* resulted in a greater sensitivity to ABA (Figure 6), thereby implying that *SaRCC1* might be involved in the response to salinity stress via ABA-dependent signaling pathways. Besides, salinity induces osmotic stress, which compromises the ability of plants to take up water [2]. However, compared with WT plants, the transgenic plants overexpressing *SaRCC1* produced no significant difference in response to 200 mM mannitol stress (Figure S4), indicating that *SaRCC1* reduces the plant resistance to salinity not by affecting the metabolic regulation of intracellular osmotic substances.

The expression of stress-responsive genes presents different expression patterns under salinity stress, ABA treatment, and osmotic stress conditions. The overexpression of *SaRCC1* decreased the salinity tolerance in *Arabidopsis* by down-regulating the expression of some stress-responsive genes including *RD29B*, *COR15A*, and *P5CS1*, thus *SaRCC1* may respond to salinity stress by regulating these stress-related genes. However, some stress-related genes such as *RD29A* and *COR47* were highly induced in *SaRCC1*-overexpressing transgenic lines upon ABA treatment, suggesting a complex regulating pathway affected by *SaRCC1* overexpression (Figure 7). However, how *SaRCC1* functions as a negative protector during salinity stress should be further investigated in detail.

In conclusion, we identified an RCC1 family protein SaRCC1 in *S. alterniflora* and investigated its biological function. Our results indicate that *SaRCC1* is induced by salinity stress. SaRCC1-overexpressing lines exhibited an increased sensitivity to salinity stress and exogenous ABA treatment and reduced the resistance to salinity stress. Our results also indicated that *SaRCC1* negatively regulates the salinity stress response by regulating stress-related genes.

## 4. Materials and Methods

### 4.1. Plant Materials and Treatments

*S. alterniflora* seedlings were grown in plastic pots containing a soil and vermiculite mixture (3:1, *v*/*v*), and then placed inside a plant growth chamber with 14 h light and 10 h dark at 25/22 °C day/night temperatures, as previously reported [21]. For full-length cDNA library construction, uniform seedlings (4- to 5-leaf stage with six plants per pot) were treated with 100 mM and 500 mM NaCl (20 pots per treatment), respectively. Afterwards, the middle parts of the second and third leaves were harvested at 0.5, 1, 2, 4, 12, and 24 h, as well as 1, 2, 3, and 4 weeks. For gene expression analysis, four- to five-leaf stage uniform seedlings of *S. alterniflora* were treated with different concentrations of NaCl (0, 100, 200, 400, and 800 mmol L^−^^1^). Then, the leaves were collected at 24, 48, and 72 h from at least three representative plants. All of the samples were frozen immediately in liquid nitrogen and then preserved at −80 °C.

The *Arabidopsis thaliana* Columbia ecotype (Col-0) were grown under long-day conditions with a photoperiod of 16 h light/8 h dark at 22 °C. For the germination and root growth analysis, wild-type (WT) and transgenic *Arabidopsis* seeds were surface sterilized and then placed on 1/2 Murashige and Skoog (MS) solid medium (pH 5.8) with 150 mM NaCl for the salinity stress, with 0.5 μM ABA for the ABA-response experiment, and with 200 mM mannitol for the osmotic stress experiment. After cold acclimation at 4 °C for 3 days, the plates were transferred into a growth chamber. Germination or green cotyledon rate was scored and primary root length was calculated with ImageJ software at the indicated time after transferring. For stress-related maker gene expression analysis, 7-day-old *Arabidopsis* seedlings from WT and transgenic lines were transferred into 150 mM NaCl, 5 μM ABA, or 300 mM mannitol for 1 d, and then subjected to determine the transcript changes of target genes.

For UV-B irradiation treatment, *Arabidopsis* seeds were germinated and grown under constant weak white light (Philips TLD18W/54-765, 6 μmol m^−2^ s^−1^, measured by AS823 Radiometer Photometer) with supplemental narrowband UV-B (Philips TL20W/01RS narrowband UV-B tubes, 2 W/m^2^, measured by LUYOR-340 UV Light Meter), as previously described [34]. The UV-B light was filtered through 300 nm transmission cutoff filters ZJB300 (+UV-B) or 340 nm cutoff filters ZJB340 (-UV-B).

### 4.2. The Isolation of SaRCC1 and Sequence Analysis

The salt-induced cDNA library of *S.* *alterniflora* was constructed using the Creator™ SMART™ cDNA Library Construction Kit, as described previously [21]. Then, a full-length cDNA homologous to the RCC1 family protein gene (namely *SaRCC1*) was isolated. Specific primers were designed and synthesized to amplify the open reading frame (ORF) fragment of *SaRCC1* (Table S1). The PCR product was purified and cloned into pMD19-T vector for sequencing.

Sequence alignment analyses were conducted using DNAMAN software. The conserved domains of presumed proteins were analyzed with Expasy online software (http://www.prosite.expasy.org/scanprosite, accessed on 6 May 2022). Additionally, different plant RCC1 family proteins (Table S2) were analyzed with MEGA software (version 5.0) for phylogenetic, using the maximum likelihood method (1000 bootstrap replicates) described previously [35].

### 4.3. RNA Isolation, Semi-Quantitative Reverse Transcription PCR (RT-PCR), and Quantitative Real-time PCR (qRT-PCR)

The total RNA was extracted from *S. alterniflora* and *Arabidopsis* seedlings using RNAprep Pure Plant Kit (Tiangen Biotech, Beijing, China) and purified with RNase-free DNase I (Tiangen Biotech, Beijing, China). After checking the integrity and quality, one microgram of total RNA was used to synthesize the first strand cDNA using the PrimeScript^TM^ RT Reagent Kit with gDNA Eraser (Perfect Real Time, TaKaRa Bio Inc., Dalian, China). The cDNA templates were then used for RT-PCR or qRT-PCR amplification. *Tubulin* gene of *S. alterniflora* and *Actin2* gene of *Arabidopsis* was used as an internal reference, and the 2^-ΔCt^ method was presented as relative expression levels of the genes analyzed by qRT-PCR. The gene-specific primers for *SaRCC1* and stress-related marker gene primers are listed in Table S1.

### 4.4. Subcellular Localization of SaRCC1

The full-length gene coding region of *SaRCC1* was amplified using specific primers (Table S1). The amplification product was inserted upstream of the enhanced green fluorescent protein (eGFP) coding region in the pAN580 expression vector. In addition, *Arabidopsis HMGB1* gene (At3g51880) and *PEX7* gene (At1g29260) fused with mCherry protein was constructed for the nucleus-localized marker [36] and peroxisome-localized marker [22], respectively. The transient expression of eGFP or mCherry fusion proteins in *Arabidopsis* mesophyll protoplasts was carried out as previously described [37], and then observed under a confocal laser scanning microscope (LSM900; Carl Zeiss, Germany).

### 4.5. Plasmid Construction and The Genetic Transformation of Arabidopsis

The PCR products of the *SaRCC1* ORF sequence with *Bam*HI/*Kpn*I sites were inserted into the pCHF1301 vector in sense or antisense orientation, respectively. The recombinant construction carrying *SaRCC1* gene was then converted into *Agrobacterium* strain EHA105, and the transformation was performed in *Arabidopsis* plants via the floral dip method [38]. The initial T_0_ transgenic lines carrying the sense-*SaRCC1* or antisense-*SaRCC1* constructs were selected on 1/2 MS solid medium supplemented with 25 mg/L hygromycin, and then detected by expression by PCR analysis using specific primers (Table S1). The representative homozygous T_3_ generation seeds obtained were used for further investigation.

### 4.6. Statistical Analysis

Data were presented as means ± standard deviation (SD) of three independent experiments. Differences among treatments were analyzed by one-way analysis of variance (ANOVA) with Duncan’s multiple range test or *t*-test (*p* < 0.05).

## Data Availability

All data in the manuscript are available from the corresponding author upon request.

## Supplementary Materials

The supporting information can be downloaded at https://www.mdpi.com/article/10.3390/ijms23158172/s1.
