# Supplementary material for: SaRCC1, a Regulator of Chromosome Condensation 1 (RCC1) Family Protein Gene from Spartina alterniflora, Negatively Regulates Salinity Stress Tolerance in Transgenic Arabidopsis
Source: Int J Mol Sci. 2022 Jul 25;23(15):8172. doi: 10.3390/ijms23158172 (PMC9332369; doi:10.3390/ijms23158172)

# Supplementary Materials for

# *SaRCC1*, a Regulator of Chromosome Condensation 1 (RCC1) Family Protein Gene from *Spartina alterniflora*, Negatively Regulates Salinity Stress Tolerance in Transgenic *Arabidopsis*

**Table S1** List and information of primers used in this study.

| Primer names | Forward primer (5'–3') | Reverse primer (5'–3') | usage |
|---|---|---|---|
| *AtActin2* | CTTCCTCAATCTCATCTTCTTCCGCTC | ATACCTCTCTTGGATTGTGCTTCATC | Semi- and qRT-PCR normalization |
| *AtRCC1-2* | GTCTCGATGGTGTGTCCATGAA | TCAGTTGGGATGACTAAAACCGGG | Semi RT-PCR expression |
| *qAtRCC1-2* | GAGAAATCATTGGTGAAGTAGCTCC | ACGCGAATTGGCACCAGCGGGGC | qRT-PCR expression |
| *SaTubulin* | GAAGGTGATGAGGGTGATGAGT | TTCAAGCAAACAAGCCTTCATA | qRT-PCR normalization |
| *SaRCC1* | AAAGGAGCCCAAGAAGGTCAAGGCAT | GCTACAACATGAACAGCTCCACAAGA | Semi RT-PCR expression |
| *qAtUVR8* | TACTGGTGGTGAGAAAATGTCAATGGTT | GTCAATGCCATTGTATGTCTCCAACCT | Semi- and qRT-PCR expression |
| *qSaRCC1* | ATGACGTGCACTGCAACCTCCGTGT | TGAGGCTGTCTGCATTCCTCAATTTG | qRT-PCR expression |
| *HPT* | CTCTGATAGAGTTGGTCAAGAC | GATATGTCCTGCGGGTAAATAG | PCR verification for transgenic plants |
| *SaRCC1-sense* | CGAAGGATCCATGGATATAGATGACGTG | CTAGGGTACCTCTGTCAGTTGGTTAACTGGC | *35S*: *SaRCC1* overexpression |
| *SaRCC1-antisense* | GCTCGGTACCATGGATATAGATGACGTGC | TAGAGGATCCTCTGTCAGTTGGTTAACTGGC | *35S*: Antisese-*SaRCC1* overexpression |
| *SaRCC1-GFP* | CTTAAGTCCGGAGCTAGCTCTAGAATGGATATAGATGACGTGC | CTCGCCCTTGCTCACCATGGATCCACTGGCTTTTCCCTGAACT | Subcellular localization |
| *AtHMGB1-mCherry* | CGCTTCTAGAATGGCTGACAAGAAGATTAG | AATCCCCGGGGGCCTTTGACATGTGGACGAT | nucleus-localized marker |
| *AtPEX7-mCherry* | CGCTTCTAGAATGCCGGTGTTCAAAGCTCC | AATCCCCGGGACTGGCTCTAGGATCCATCCC | peroxisome-localized marker |
| qAtHY5 | ACAAGCGGCTGAAGAGGTTGTTG | TCTGGTTCTCGTTCTGAAGAGT | qRT-PCR expression |
| AtRD29A | TCTGAAGAACGAATCTGATAT | GCCGGAAATTTATCCTCTTCTGA | qRT-PCR expression |
| AtRD29B | TACATCAGCCATAACTAACAAA | TGCCCGTAAGCAGTAACAGATC | qRT-PCR expression |
| AtCOR47 | AGTGAAGGAGAACAAGATTACTC | TCTTCATCGCTCGAAGAGGAAG | qRT-PCR expression |
| AtCOR15A | AACGAGGCCACAAAGAAAGC | CAGCTTCTTTACCCAATGTATCTGC | qRT-PCR expression |
| AtRD22 | CACCGATGCAGAAGTACAAAAT | CCGTTCTCTCCCTCGAGCGGAA | qRT-PCR expression |
| AtABI2 | GGGAAAGTAATCCGGTGGAACGG | TAAGATGAGACAATCATCTTCTT | qRT-PCR expression |
| AtP5CS1 | TCACCGACATGGGAGTGCACACAC | TGCCCGTGCTTACCCCCACCTCT | qRT-PCR expression |

**Table S2** Accession numbers of UVR8 and RCC1 proteins used in Fig. 1.

| Protein names | Species | Accession number | References |
|---|---|---|---|
| SaRCC1 | *Spartina alterniflora* | This study | |
| SbUVR8 | *Sorghum bicolor* | XP_021307437.1 | NCBI |
| DoUVR8 | *Dichanthelium oligosanthes* | OEL17715.1 | NCBI |
| SiUVR8 | *Setaria italica* | XP_004982469.1 | NCBI |
| AtaRUG3 | *Aegilops tauschii subsp. strangulata* | XP_020146593.1 | NCBI |
| BdUVR8 | *Brachypodium distachyon* | XP_003562310.1 | NCBI |
| ObUVR8 | *Oryza brachyantha* | XP_006650287.1 | NCBI |
| CsUVR8 | *Cannabis sativa* | XP_030485099.1 | NCBI |
| OeUVR8 | *Olea europaea var. sylvestris* | XP_022884180.1 | NCBI |
| PdUVR8 | *Phoenix dactylifera* | XP_008794204.2 | NCBI |
| EgUVR8 | *Elaeis guineensis* | XP_010938025.1 | NCBI |
| PaUVR8 | *Prunus avium* | XP_021816775.1 | NCBI |
| PpeUVR8 | *Prunus persica* | XP_007217882.1 | NCBI |
| PduUVR8 | *Prunus dulcis* | XP_034208971.1 | NCBI |
| ZmRCC1 | *Zea mays* | NP_001159191.1 | NCBI |
| AtPROTON1 | *Arabidopsis thaliana* | AT1G19880.1 | Tair |
| AtSAB1 | *Arabidopsis thaliana* | AT1G27060.1 | Tair |
| AtPARF-1 | *Arabidopsis thaliana* | AT1G65920.1 | Tair |
| AtRCC1-1 | *Arabidopsis thaliana* | AT1G69710.1 | Tair |
| AtPRAF1 | *Arabidopsis thaliana* | AT1G76950.1 | Tair |
| AtRCC1-2 | *Arabidopsis thaliana* | AT3G02300.1 | Tair |
| AtRCC1-3 | *Arabidopsis thaliana* | AT3G02510.1 | Tair |
| AtRCC1-4 | *Arabidopsis thaliana* | AT3G03790.3 | Tair |
| AtRCC1-5 | *Arabidopsis thaliana* | AT3G15430.1 | Tair |
| AtRCC1-6 | *Arabidopsis thaliana* | AT3G23270.1 | Tair |
| AtRCC1-7 | *Arabidopsis thaliana* | AT3G26100.2 | Tair |
| AtRCC1-8 | *Arabidopsis thaliana* | AT3G47660.1 | Tair |
| AtRCC1-9 | *Arabidopsis thaliana* | AT3G53830.1 | Tair |
| AtTCF1 | *Arabidopsis thaliana* | AT3G55580.1 | Tair |
| AtRCC1-10 | *Arabidopsis thaliana* | AT4G14368.1 | Tair |
| AtRUG1 | *Arabidopsis thaliana* | AT5G08710.1 | Tair |
| AtRCC1-11 | *Arabidopsis thaliana* | AT5G11580.1 | Tair |
| AtRCC1-12 | *Arabidopsis thaliana* | AT5G12350.1 | Tair |
| AtRCC1-13 | *Arabidopsis thaliana* | AT5G16040.1 | Tair |
| AtRCC1-14 | *Arabidopsis thaliana* | AT5G19420.2 | Tair |
| AtRCC1-15 | *Arabidopsis thaliana* | AT5G42140.1 | Tair |
| AtRUG2 | *Arabidopsis thaliana* | AT5G48330.1 | Tair |
| AtRUG3 | *Arabidopsis thaliana* | AT5G60870.1 | Tair |
| AtUVR8 | *Arabidopsis thaliana* | AT5G63860.1 | Tair |
| AtUVR1 (AtUVH3) | *Arabidopsis thaliana* | AT3G28030.1 | Tair |
| AtUVR2 | *Arabidopsis thaliana* | AT1G12370.2 | Tair |
| AtUVR3 | *Arabidopsis thaliana* | AT3G15620.1 | Tair |
| AtERCC1 (AtUVR7) | *Arabidopsis thaliana* | AT3G05210.1 | Tair |
| BpUVR8 | *Betula platyphylla* | AHY02156.1 | Li et al., 2018 |
| CmUVR8 | *Chrysanthemum morifolium* | | Yang et al., 2018 |
| CqUVR8 | *Colobanthus quitensis* | QEM23329.1 | Contreras et al., 2019 |
| CrUVR8 | *Chlamydomonas reinhardtii* | AKS29686.1 (Cre05.g230600) | Tilbrook et al., 2016 |
| MdUVR8 | *Malus domestica* | KU095866 | Zhao et al., 2016 |
| MpUVR8.1 | *Marchantia polymorpha* | Mapoly0023s0125.1 | Soriano et al., 2018 |
| MpUVR8.2 | *Marchantia polymorpha* | Mapoly0023s0125.2 | Soriano et al., 2018 |
| OsUVR8a | *Oryza sativa Japonica Group* | Os02g0554100, | Idris et al., 2020 |
| OsUVR8b | *Oryza sativa Japonica Group* | Os04g0435700 | Idris et al., 2020 |
| PeUVR8 | *Populus euphratica* | AKJ54489.1 | Mao et al., 2015 |

| | | | |
|---|---|---|---|
| PpUVR8.1 | *Physcomitrella patens* | Pp3c10_2230 | Soriano et al., 2018 |
| PpUVR8.2 | *Physcomitrella patens* | Pp3c3_15300 | Soriano et al., 2018 |
| SlUVR8 | *Solanum lycopersicum* | Solyc05g018620 | Li et al., 2018 |
| VvUVR1 | *Vitis vinifera* | VIT_07s0031g02560 | Loyola et al., 2016 |
| ZmDEK47 (ZmRUG3) | *Zea mays* | NP_001144762.1 | Cao et al., 2021 |
| ZmUVR8 | *Zea mays* | GRMZM2G003565 | Fernández et al., 2020 |

**Figure S1** Nucleotide and predicted peptide sequence of the *SaRCC1* gene. ORF is shown in upper-case letters; 3'- and 5'-UTRs are in lower-case letters. The predicted amino acids are displayed with single-letter symbols below their capitalized codon using DNAMAN.

```
-121                                                      gacgaggacaaaggaacaaggaggcaactccattccgcaggtcaaa
        agaggagaggagcactcaaagctccgacctttccaggaatcctcccggaagattgcaccattctctccaggggtc
1       ATGGATATAGATGACGTGCACTGCAACCTCCGTGTTGTTGGTGTGCCAACGAAAAGTGCAATTTACATATGGGGC
1        M  D  I  D  D  V  H  C  N  L  R  V  V  G  V  P  T  K  S  A  I  Y  I  W  G
76      TATAACCAGAGTGGGCAGACGGCACGAAAGGGCAAGGAATGCTACTTGAGGATTCCCAAGAGCCTCCCTCCCAAG
26       Y  N  Q  S  G  Q  T  A  R  K  G  K  E  C  Y  L  R  I  P  K  S  L  P  P  K
151     CTGTTCAAATTGAGGAATGCAGACAGCCTCAGGTGGACTGACATTGCATGTGGCCGTGAGCACACTGCTGCAGTT
51       L  F  K  L  R  N  A  D  S  L  R  W  T  D  I  A  C  G  R  E  H  T  A  A  V
226     GCTTCTGACGGATCACTCTTCACCTGGGGTGCTAATGAGTTTGGCCAGTTGGGAGATGGTACAGAAAAGAGTTCA
76       A  S  D  G  S  L  F  T  W  G  A  N  E  F  G  Q  L  G  D  G  T  E  K  S  S
301     AAGGAGCCCAAGAAGGTCAAGGCATTGGAGACTGAATTTGTGAAATCAGTATCCTGCGGTGCACATTGTACAGCT
101      K  E  P  K  K  V  K  A  L  E  T  E  F  V  K  S  V  S  C  G  A  H  C  T  A
376     GCCATTGCTGAGCCTCGAGAAAGTGATGGAACAGTATCAAAAAACAGGCTTTGGGTTTGGGGACAAAATCAGGGT
126      A  I  A  E  P  R  E  S  D  G  T  V  S  K  N  R  L  W  V  W  G  Q  N  Q  G
451     TCAGACAACCCTCGCCTATTCTGGGGAGATTTTACACCAAACACAGTGATCCAGCAGGTTTCTTGTGGAGCTGTT
151      S  D  N  P  R  L  F  W  G  D  F  T  P  N  T  V  I  Q  Q  V  S  C  G  A  V
526     CATGTTGTAGCTTTATCCGAAGATGGTCTCCTGCAAGCTTGGGGGTACAATGAGTACGGTCAGCTTGGCAGAGGT
176      H  V  V  A  L  S  E  D  G  L  L  Q  A  W  G  Y  N  E  Y  G  Q  L  G  R  G
601     TGTACTTCTCAAGGACTTCAGGGAGCTCGTGTACTAAATGCTTATGCAAGATTCCTTGATGATGCTCCAGAGCTA
201      C  T  S  Q  G  L  Q  G  A  R  V  L  N  A  Y  A  R  F  L  D  D  A  P  E  L
676     GTGAAGATTGTTAGGGTGTCATGTGGAGAGTACCATACAGCAGCTATATCAAAAAATGGGGAGGTATATACCTGG
226      V  K  I  V  R  V  S  C  G  E  Y  H  T  A  A  I  S  K  N  G  E  V  Y  T  W
751     GGACTGGGAAGCATGGGGCAGCTTGGGCATTGCTCCCTGCAGTCTGGAGACAAGGAGCTAATCCCTAGGCGAGTT
251      G  L  G  S  M  G  Q  L  G  H  C  S  L  Q  S  G  D  K  E  L  I  P  R  R  V
826     GTTGCCCTTGATGGGATAATAGTCCGAGACGTATCTTGTGGAGGTGTCCACTCTTGTGCTGTGACTCAAAATGGA
276      V  A  L  D  G  I  I  V  R  D  V  S  C  G  G  V  H  S  C  A  V  T  Q  N  G
901     GCTTTGTATGCTTGGGGCGGAGGACACGTGGGTCAGCTGGGAGTTGGACCTCAGAGTTGCTTCTTTTCTTGCTCT
301      A  L  Y  A  W  G  G  G  H  V  G  Q  L  G  V  G  P  Q  S  C  F  F  S  C  S
976     CTTAATGGATCTGAAATGCTGCTACGCAACATTCCAGTCCTGGTCATACCGTCAGGTGTCCGTCTTGCTACCTGT
326      L  N  G  S  E  M  L  L  R  N  I  P  V  L  V  I  P  S  G  V  R  L  A  T  C
1051    GGACACTCTCACACACTTGTATCTATGAAAGATGGCCGTATATACGGGTGGGGCTATAATAGTTATGGCCAGGCA
351      G  H  S  H  T  L  V  S  M  K  D  G  R  I  Y  G  W  G  Y  N  S  Y  G  Q  A
1126    GCAAACGAGAAGTCAACTTACGCTTGGTTCCCCTCTCCAGTTGATTGGTGTGTTGGTGAGGTGAGAAGACTTGCT
376      A  N  E  K  S  T  Y  A  W  F  P  S  P  V  D  W  C  V  G  E  V  R  R  L  A
1201    GCTGGAGGTGGCCATTCAGCTGTACTGACTGATGCATGTTCATTGAAGGAGCTGTGTGAGTTCAAGCTGGCAGAG
401      A  G  G  G  H  S  A  V  L  T  D  A  C  S  L  K  E  L  C  E  F  K  L  A  E
1276    ACTGTAAACATTTCTAACGCTCAGCTAATAGAAGATGTTGCGTCACGGACTGGTGCTGATGCCTTGGCCCGTTTA
426      T  V  N  I  S  N  A  Q  L  I  E  D  V  A  S  R  T  G  A  D  A  L  A  R  L
1351    TGTGAGAAATTAAGGGAACATCCGGATGTGCAAGGAGAATGTGAATTCCTCGAAAAGCAAGTAGATGGAGTTCAG
451      C  E  K  L  R  E  H  P  D  V  Q  G  E  C  E  F  L  E  K  Q  V  D  G  V  Q
1426    GGAAAAGCCAGTTAAccaactgacagatacttgacagtttgaggtatcggccattaatagttattgtggcacttg
476      G  K  A  S  *
ctgtctttaacggtgccatgttattgattcagacattaattgttgtagtcatggcagattgaacagtgtcaatggacttggg
acctggtttttattggtgtataaaccgattggtgtgtctcccatgtgtatcgaaggcgtggaggccctgtgtagatcatgaa
cttagtgtacttttgttgaactaaatggaaacttgtgcctgggtgaaaaaaaaaaaaaaaaaaaaaaaaaaa    +298
```

**Figure S2** Nucleotide sequence alignment between *SaRCC1* and *AtRCC1-2*. The alignment was performed using DNAMAN software. Primers used for semi RT-PCR qRT-PCR in *SaRCC1* and *AtRCC1-2* were shown by blue arrow and red arrow, respectively.

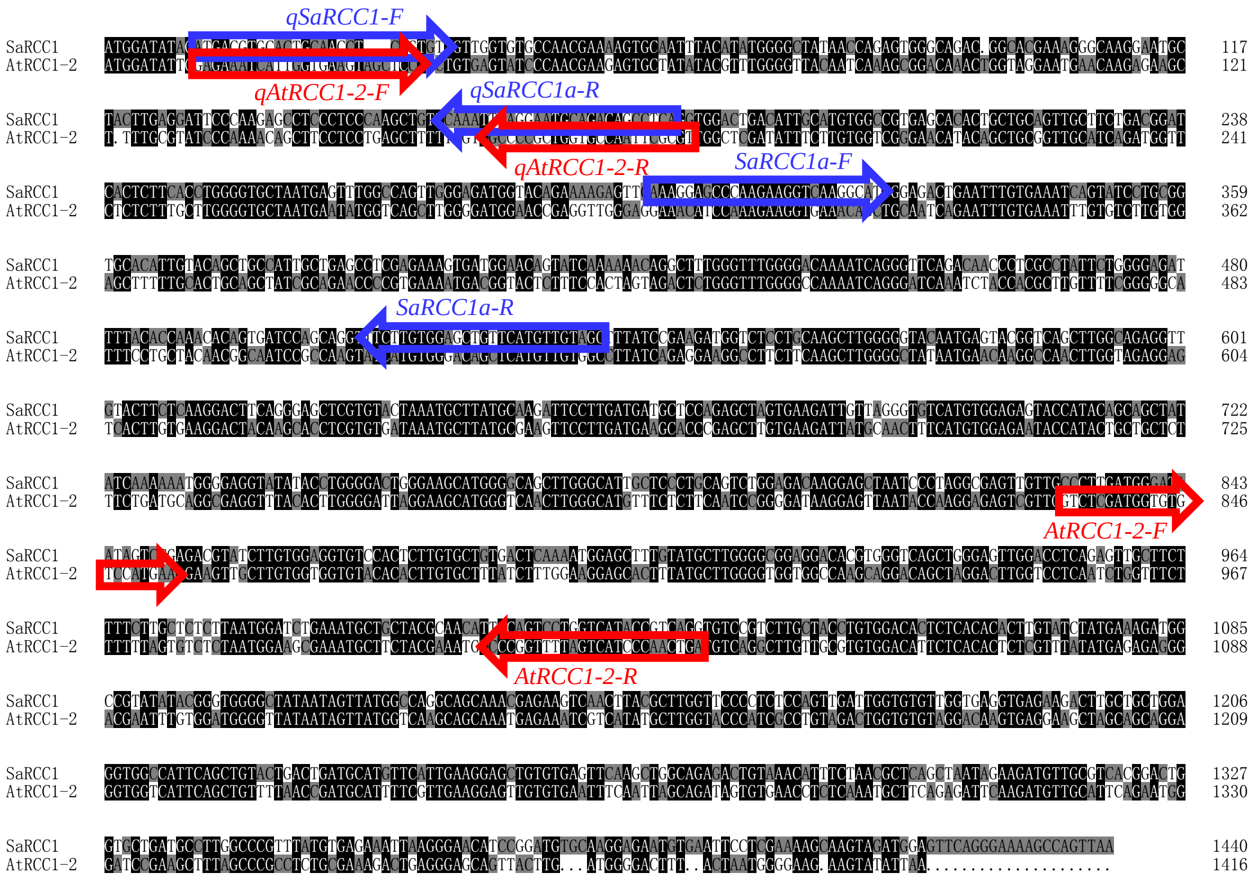

**Figure S3** Amino acid sequence alignment between *SaRCC1* and *AtUVR8*. The alignment was performed using DNAMAN software.

```
SaRCC1    ....MDIDDVHCNLRVVGVPTKSAIYIWGYNQSGQTARKGKECYLRIPKSLP...PKLFKLRNADSLRWTDIACGREHTAAV     75
AtUVR8    MAEDMAADEVTAPPRKVLIISAGASHSVALLSGDIVCSWGRGEDGQLGHGDAEDRPSPTQLSALDGHQIVSVTCGADHTVAY     82

SaRCC1    ASDG.SLFTWGANEFGQLGDGTEKSSKEPKKVKALETEFVKSVSCG.AHCTAAIAEPRESDGTVSKNRLWVWGQNQGSDNPR    155
AtUVR8    SQSGMEVYSWGWGDFGRLGHGNSSDLFTPLPIKALHGIRIKQIACGDSHCLAVTMEGEVQSWGRNQNGQLGLGDTEDSLVPQ    164

SaRCC1    LFWGDFTPNTVIQQVSCGAVHVVALSEDGLLQAWGYNEYGQLGRGCTSQGLQGARVLNAYARFLDDAPELVKIVRVSCGEYH    237
AtUVR8    KIQ..AFEGIRIKMVAAGAEHTAAVTEDGDLYGWGWGRYGNLGLGDRTDRLVPERVTSTGG.........EKMSMVACGWRH    235

SaRCC1    TAAISKNGEVYTWGLGSMGQLGHCSLQSGDKELIPRRVVALDGIIVRDVSCGGVHSCAVTQNGALYAWGGGHVGQLGVGPQS    319
AtUVR8    TISVSYSGALYTYGWSKYGQLGHGDLED...HLIPHKLEALSNSFISQISGGWRHTMALTSDGKLYGWGWNKFGQVGVGN..    312

SaRCC1    CFFSCSLNGSEMLLRNIPVLVIPSGVRLATCGHSHTLVSMKDGRIYGWGYNSYGQAANEKSTYAWFPSPVDWCVGEVRRLAA    401
AtUVR8    .....NLDQCSPVQVRFPD...DQKVVQVSCGWRHTLAVTERNNVFAWGRGTNGQLGIGESVDRNFPKIIEALSVDG...AS    383

SaRCC1    GGGHSAVLTDACSLKELCEFKLAETVNISNAQLIEDVASRTGADALARLCEKLREHPDVQGECEFLEKQVDGVQGKAS        479
AtUVR8    GQHIESSNIDPSSGKSWVSPAERYAVVPDETGLTDGSSKGNGGDISVPQTDVKRVRI.....................        440
```

**Figure S4** Construction of transgenic *Arabidopsis* for overexpression and antisense-overexpression of *SaRCC1*. (A) Construct of *SaRCC1* used in this study. (B) PCR analyses were performed on transgenic $T_2$ *Arabidopsis* plants, using *HPTII* gene primer. (C) Semi-quantitative reverse transcription PCR (RT-PCR) analysis of *SaRCC1*, *AtUVR8* and *AtRCC1-2* expression in transgenic *Arabidopsis* plants. RT-PCR was performed using RNA extracted from wild-type (WT), five independent *SaRCC1*-overexpressing, and ten independent *SaRCC1*-antisense-overexpressing lines. Specific primer pairs were used to detect their transcripts. Primers specific for *Arabidopsis* actin (*AtActin2*) were used for the loading control. *hyg*: hygromycin-phosphotransferase gene; *35S*: Cauliflower mosaic virus (CaMV) 35S promoter; Tnos: nopaline-synthase terminator; LB: T-DNA left border; RB: T-DNA right border. M, 250-bp DNA ladder marker; WT, wild-type *Arabidopsis* plants; N, negative control ($dH_2O$ were used as PCR template).

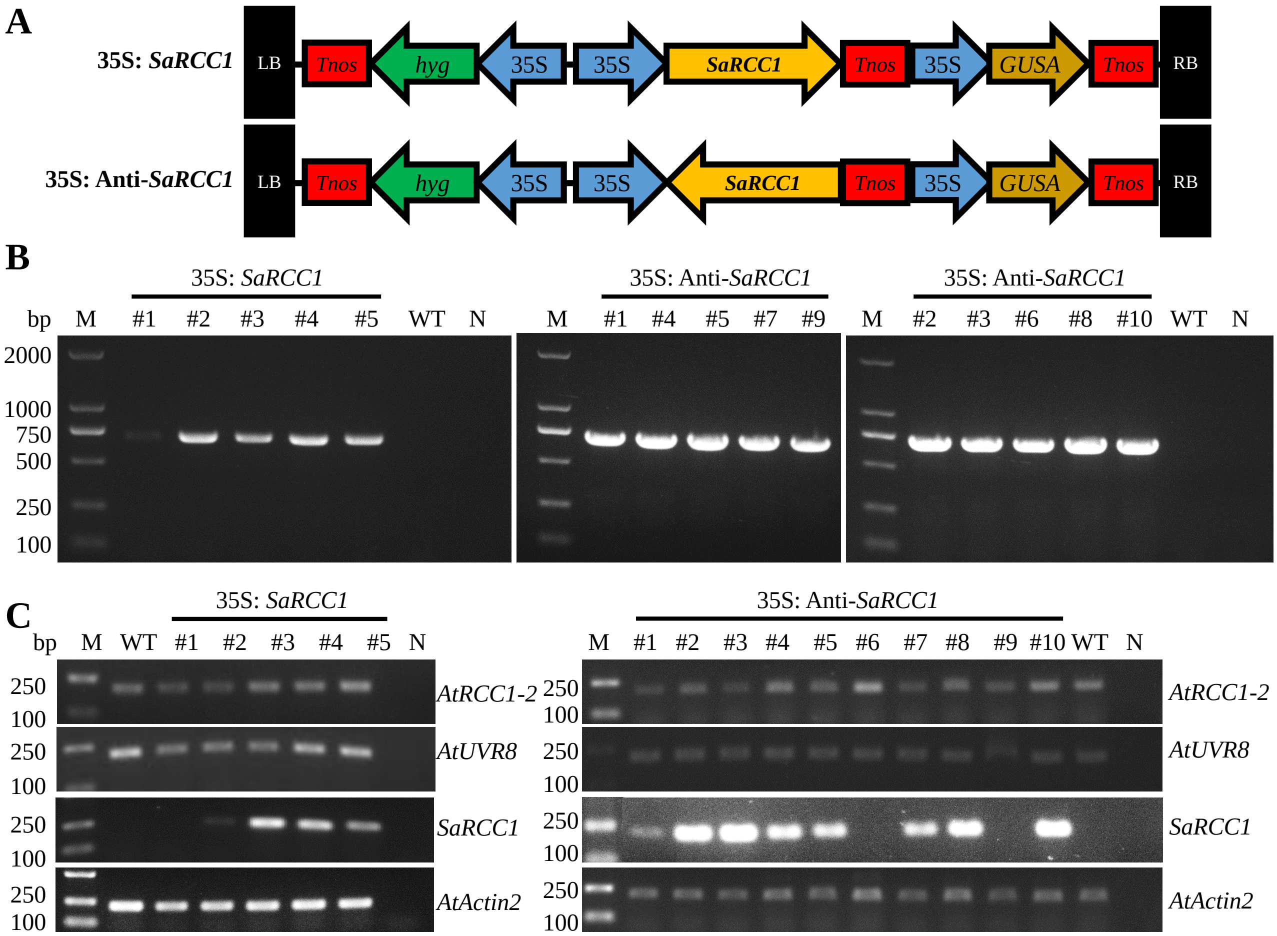

**Figure S5** Analysis of osmotic tolerance in *Arabidopsis* wild-type (WT) and *SaRCC1* overexpressing and antisense-overexpressing plants. The phenotype (A), germination rates (B), and root length (**C**) of wild-type (WT) and *SaRCC1* overexpressing and antisense-overexpressing *Arabidopsis* in 1/2 MS medium with or without 200 mM mannitol for 7 d. Different letters indicate significant differences ($p < 0.05$) according Duncan's multiple test.

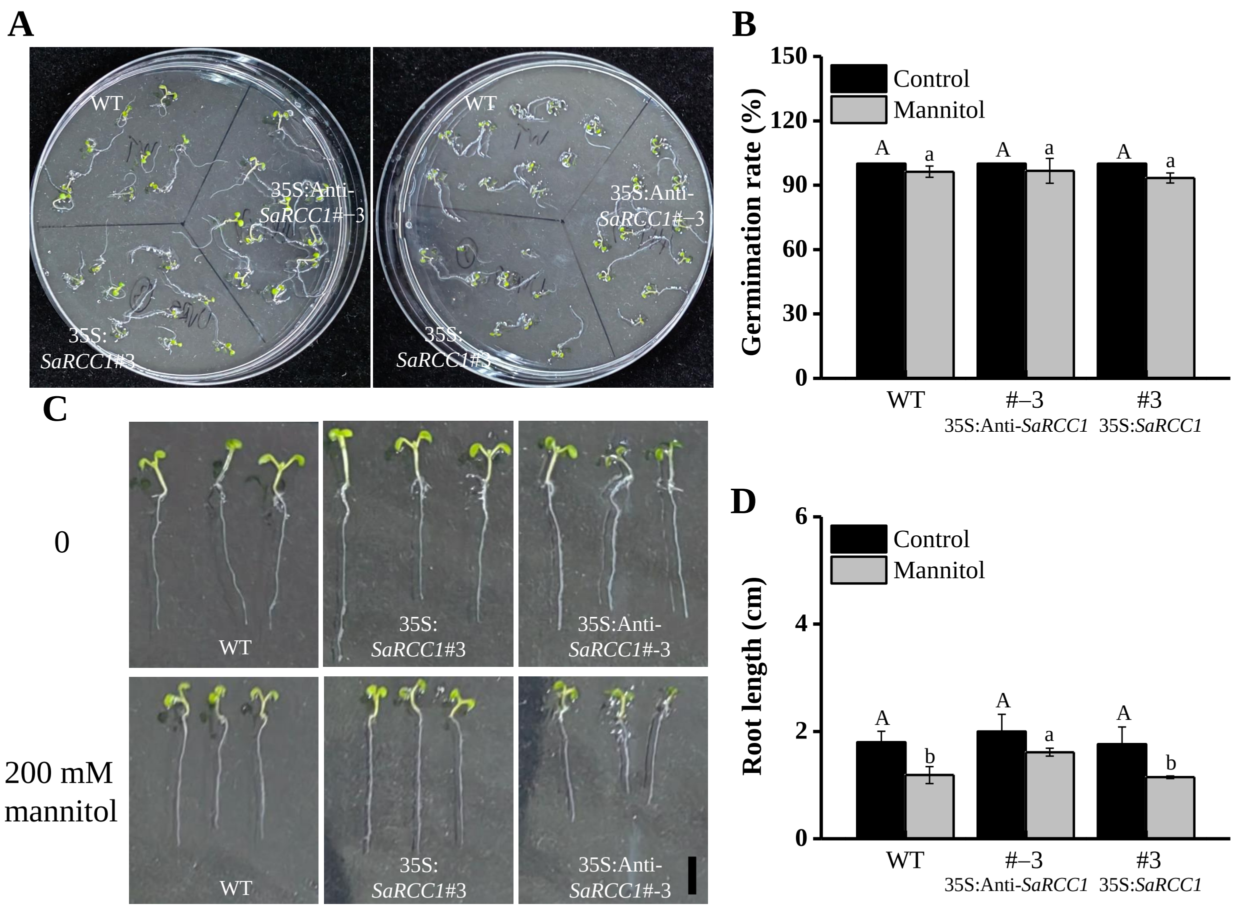

**Figure S6.** Expression levels of *SaRCC1*, *AtUVR8*, and *AtRCC1-2* genes in wild-type (WT) and transgenic plants under salinity stress, ABA treatment, and osmotic stress conditions. Seven-day-old seedlings were subjected to 100 mM NaCl, 5 μM ABA, or 300 mM mannitol, respectively, 24 h, before determining their expression levels by qRT-PCR. The *actin2* gene was used as an internal reference. The asterisks over the bars indicate the significant differences between the wild-type and *SaRCC1* transgenic *Arabidopsis* lines.

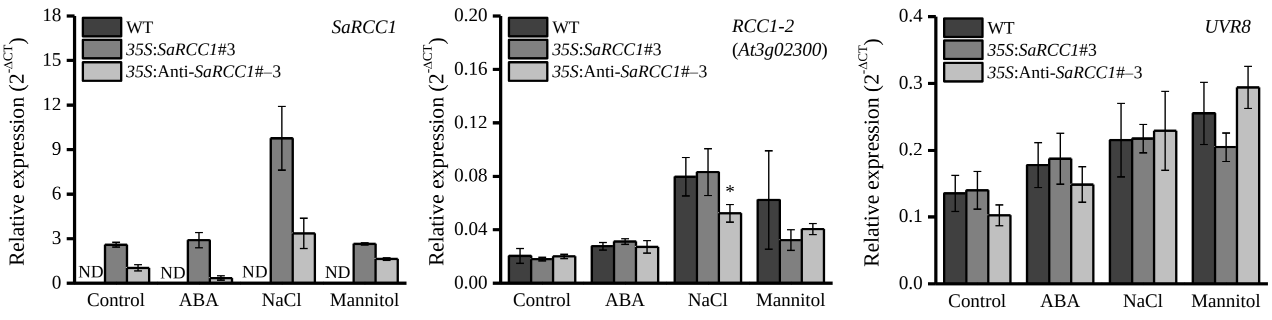

Supplement: Supplementary file 1 [file ijms-23-08172-s001.zip › ijms-1748898-supplementary.pdf]
